# Supplementary material for: Direct evidence for charge stripes in a layered cobalt oxide
Source: Nat Commun. 2016 May 23;7:11632. doi: 10.1038/ncomms11632 (PMC4879241; doi:10.1038/ncomms11632)
Supplement: Supplementary Information — Supplementary Figures 1-4, Supplementary Tables 1-2 and Supplementary Notes 1-2 [file ncomms11632-s1.pdf]

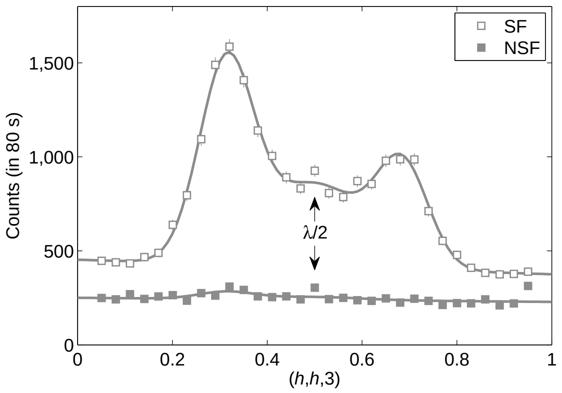

Supplementary Figure 1: Separation of magnetic and nuclear scattering. Raw polarized neutron diffraction intensities for $La_{5/3}Sr_{1/3}CoO_4$ measured at a temperature of 2 K along the line $(h, h, 3)$ in reciprocal space. The two peaks in the SF channel are from the stripe magnetic order. There is very little leakage between the two polarization channels, consistent with the assumed flipping ratio $R = 25.7$. The small peak at $h = 0.5$ in both channels is due to second order diffraction $(\lambda/2)$ from the $(1, 1, 6)$ Bragg peak. This and other peaks originating from $\lambda/2$ contamination are temperature-independent and so cancel out when scans at different temperatures are subtracted, as was done to reveal the stripe CO scattering. Error bars on the data are standard deviations obtained from neutron counts.

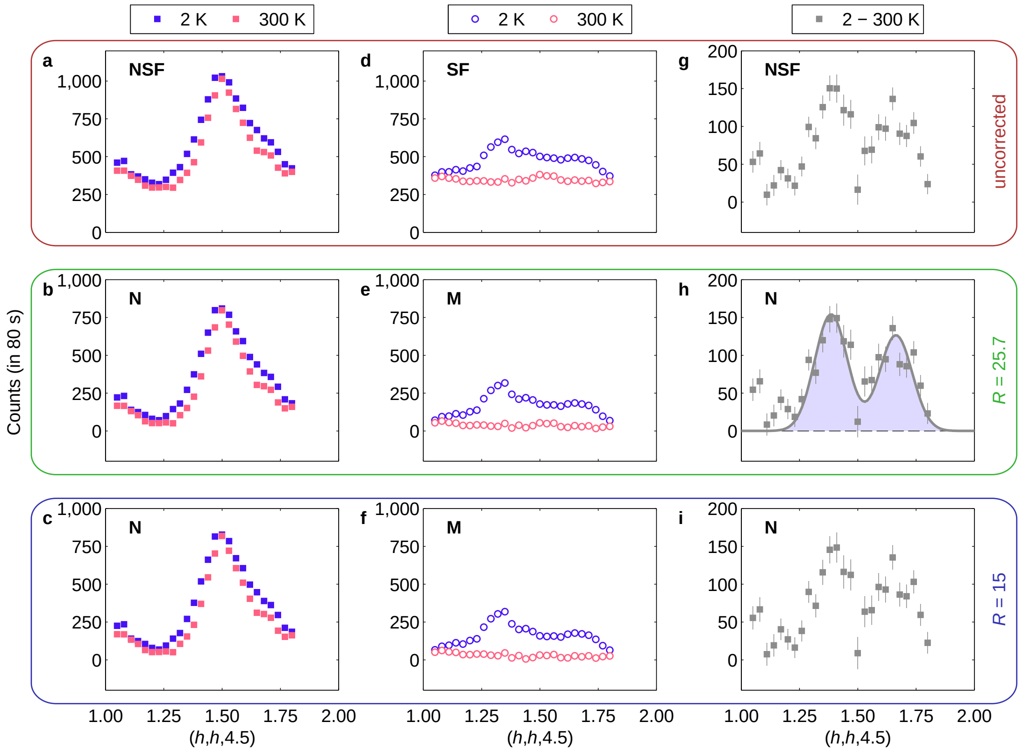

Supplementary Figure 2: Effect of polarisation corrections. The upper three panels show raw, uncorrected data. The middle and lower three panels show data corrected with $R = 25.7$ and $R = 15$, respectively. Panels **a** – **c** contain the raw NSF scattering and corrected structural diffuse scattering (N) due to checkerboard and stripe charge order, and panels **d** – **f** contain the raw SF scattering and corrected magnetic diffuse scattering (M). Panels **g** – **i** show the raw and corrected NSF signal after subtraction of the 300 K data from the 2 K data to isolate the structural diffuse scattering due to stripe charge order. Error bars on the data are standard deviations obtained from neutron counts.

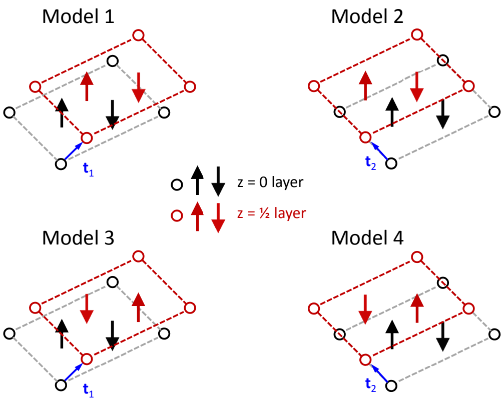

Supplementary Figure 3: Models for the spin and charge order on adjacent layers in $La_{5/3}Sr_{1/3}CoO_4$. The open circles represent Co^{3+} holes, and the arrows indicate ordered spins on Co^{2+}. Black and red symbols are for $z = 0$ and $z = 1/2$ layers, respectively. $\mathbf{t}_1$ and $\mathbf{t}_2$ are the two different stacking vectors. The parallelograms are unit cells for the spin and charge order within each CoO_2 layer.

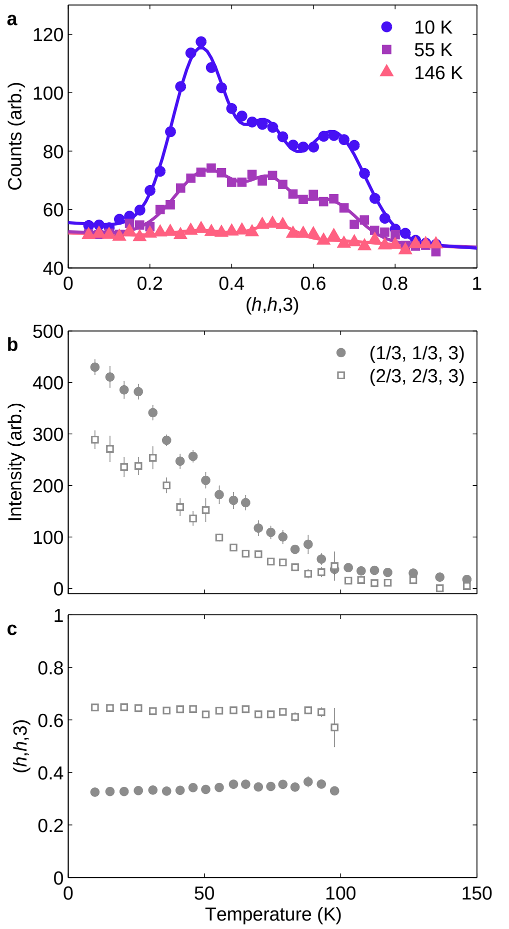

Supplementary Figure 4: Temperature dependence of magnetic order in $La_{5/3}Sr_{1/3}CoO_4$. (**a**) Unpolarised neutron diffraction measurements at 10 K, 55 K and 146 K along the line $(h, h, 3)$ in reciprocal space. (**b**) Temperature dependence of the intensity of the fitted magnetic peaks. (**c**) Temperature dependence of the magnetic peak positions. Above 100 K the magnetic signal is too weak to fit the peak centres. To estimate the magnetic intensity above 100 K the peaks centres and widths were fixed, and only their amplitudes varied.

Supplementary Table 1. Values of the factor $1 + \exp(\mathrm{i}\mathbf{Q} \cdot \mathbf{t})$ for charge order (CO) diffraction peaks for each of the four ideal period-3 stripe structure shown in Supplementary Fig. 3. Values are given for two of the CO peaks surrounding $(0.5, 0.5, l)$, one from each stripe domain.

CO	stacking	$(1/3, 1/3, l)$		$(2/3, 1/3, l)$	
model	$\mathbf{t}$	l even	l odd	l even	l odd
1 & 3	$\mathbf{t}_1$	1	3	3	1
2 & 4	$\mathbf{t}_2$	4	0	0	4

Supplementary Table 2. Values of the factor $1 + T \exp(\mathrm{i}\mathbf{Q} \cdot \mathbf{t})$ for spin order (SO) diffraction peaks for each of the four ideal period-3 stripe structures shown in Supplementary Fig. 3. Values are given for two of the SO peaks surrounding $(0.5, 0.5, l)$, one from each stripe domain.

SO	stacking	$(1/3, 1/3, l)$		$(2/3, 1/3, l)$	
model	$(\mathbf{t}, T)$	l even	l odd	l even	l odd
1	$(\mathbf{t}_1, +1)$	1	3	3	1
2	$(\mathbf{t}_2, +1)$	4	0	0	4
3	$(\mathbf{t}_1, -1)$	3	1	1	3
4	$(\mathbf{t}_2, -1)$	0	4	4	0

Supplementary Note 1: Stacking of spin and charge stripe order along c axis

In this section we analyze the stacking of the incommensurate CO and SO based on the scans presented in Fig. 2c,d in the main article, which show that spin and charge correlations extend over a short distance $\sim c$ perpendicular to the CoO_2 layers. Since both the charge and magnetic peaks are centred at integer l values the majority stacking of the (quasi-)order has the same c-axis period as the lattice. However, there are several different ways in which the order can be related on adjacent layers related by the body-centering translation.

We consider ideal site-centred period-3 stripes and assume that the magnetic structure is collinear. This means that the charge superstructure on one layer ($z = 0$) can be related to that on an adjacent layer ($z = 1/2$) by a translation $\mathbf{t}$. For the spin order, the translation is followed by the time reversal operation $T = \pm 1$, where $T = +1$ means no change in the spins, and $T = -1$ means reversal of all the spins. The structure factors for the spin and charge diffraction peaks then contain a factor $1 + T \exp(\mathrm{i}\mathbf{Q} \cdot \mathbf{t})$, where $T = +1$ for charge order and $T = \pm 1$ for spin order. In the exponential, $\mathbf{Q} \cdot \mathbf{t} = (Q_x t_x + Q_y t_y + Q_z t_z)$, where t_x, t_y, t_z are the components of $\mathbf{t}$ written as fractional coordinates along the crystallographic a, b, and c axes, and Q_x, Q_y, Q_z are the components of $\mathbf{Q}$ in reciprocal lattice units.

There are two distinct stackings of the charge order, which may be described by stacking vectors $\mathbf{t}_1 = (0.5, 0.5, 0.5)$ and $\mathbf{t}_2 = (-0.5, 0.5, 0.5)$. For each of these, the magnetic stacking can have $T = +1$ or $T = -1$. This gives four distinct superstructures for the combined spin and charge order with the periodicity of the lattice in the c direction. The unit cells of each of these are shown in Supplementary Fig. 3 for a stripe domain in which the stripes run parallel to the $[1, -1]$ direction on the square lattice. Rotation of the stripe pattern by 90° gives an equivalent domain in which the stripes are parallel to the $[1, 1]$ direction.

For ideal period-3 stripes the charge order (CO) peaks are located at $\mathbf{Q}_{\mathrm{co}} = \mathbf{G} \pm \mathbf{q}_1$ and $\mathbf{G} \pm \mathbf{q}_2$, where $\mathbf{G} = (h, k, l)$ is a reciprocal lattice vector, and the spin order (SO) diffraction peaks are located at $\mathbf{Q}_{\mathrm{so}} = \mathbf{Q}_{\mathrm{AF}} \pm \mathbf{q}_1/2$ and $\mathbf{Q}_{\mathrm{AF}} \pm \mathbf{q}_2/2$, where $\mathbf{Q}_{\mathrm{AF}} = (h + 1/2, k + 1/2, l)$ is an antiferromagnetic wavevector. $\mathbf{q}_1 = (1/3, 1/3, 0)$ and $\mathbf{q}_2 = (1/3, -1/3, 0)$ are modulation wavevectors for the two equivalent orthogonal stripe domains. In the $\mathbf{q}_1$ domain the stripes run parallel to the $[1, -1]$ direction on the square lattice, and in the $\mathbf{q}_2$ domain the stripes are parallel to the $[1, 1]$ direction.

Supplementary Tables and list the values of the $1 + T \exp(\mathrm{i}\mathbf{Q} \cdot \mathbf{t})$ factor for the quartets of CO and SO satellites surrounding $(0.5, 0.5, l)$, l even and odd. The satellite peaks $(1/3, 1/3, l)$ and $(2/3, 1/3, l)$ are representative of the two domains. The data recorded along scan B presented in Fig. 2c in the main

article show that for the $(1/3, 1/3, l)$-type peaks the SO diffuse scattering is strongest for odd l integers. From Supplementary Table one can see that this observation favours the combined spin and charge stripe stacking described by either model 1 or model 4. Scan B in Fig. 2d indicates that the CO diffuse scattering has maxima close to odd l and minima at even l, which from Supplementary Table favours models 1 and 3. Taken together, therefore, the evidence supports model 1. However, this conclusion should be considered tentative as the peaks are very broad in l and it is possible that the observed l-dependence comes entirely from the form factor of the CoO_6 distortion pattern, and not from any inter-layer correlations. A direct way to check this would be to measure the l-dependence along $(2/3, 1/3, l)$. If inter-layer correlations are significant then the non-magnetic scattering along $(2/3, 1/3, l)$ should be different from that along $(1/3, 1/3, l)$ in accord with the structure factors given in Supplementary Table . Temperature difference data to fully isolate the l-dependence of the stripe CO scattering from the tails of the checkerboard CO peaks would also be valuable.

Supplementary Note 2:
Temperature dependence of magnetic order

The temperature dependence of the magnetic diffraction peaks was studied by unpolarized neutron diffraction on the IN8 triple-axis spectrometer at the Institut Laue–Langevin. Incident and scattered neutrons with energy 14.7 meV were selected by Bragg reflection from a Si (111) monochromator and a pyrolytic graphite (002) analyzer, respectively. The resolution will be slightly different from that achieved on IN20 owing to the different monochromator and analyzer materials and the fact that both were doubly focussed on IN8 whereas the IN20 analyser was only focussed horizontally.

Scans along $(h, h, 3)$ were performed at a series of temperatures between 10 K and 146 K. Examples are shown in Supplementary Fig. 4a. From the polarized neutron measurements (Supplementary Fig. 1) there is very little structural diffuse scattering in this scan, so the temperature dependence of the scattering can be assumed to be from the magnetic order. The temperature dependence of the peak intensities and positions are shown in Supplementary Figs. 3b and 3c. These were obtained by fitting a lineshape comprising three Gaussians to the data. By 100 K the magnetic diffraction intensity has dropped to below 10% of its value at the lowest temperature but the peak positions have shifted only slightly, towards the antiferromagnetic wavevector at the centre of the scan. This indicates that the incommensurate magnetic order is coupled to the incommensurate charge order, which is almost temperature independent below 100 K (see Fig. 5 in the main article).
